# Omega-3 Polyunsaturated Fatty Acid: A Pharmaco-Nutraceutical Approach to Improve the Responsiveness to Ursodeoxycholic Acid

**DOI:** 10.3390/nu13082617

**Published:** 2021-07-29

**Authors:** Ariane Thérien, Anna Cieślak, Mélanie Verreault, Martin Perreault, Jocelyn Trottier, Stéphane Gobeil, Marie-Claude Vohl, Olivier Barbier

**Affiliations:** 1 CHU de Québec Research Center, Québec, QC G1V 4G2, Canada; ariane.therien.2@ulaval.ca (A.T.); anna.anita.cieslak@gmail.com (A.C.); melanie.verreault@crchudequebec.ulaval.ca (M.V.); martin.perreault2@ulaval.ca (M.P.); jocelyn.trottier@crchudequebec.ulaval.ca (J.T.); stephane.gobeil@crchudequebec.ulaval.ca (S.G.); 2 Faculty of Pharmacy, Université Laval, Québec, QC G1V 0A6, Canada; 3 Faculty of Medicine, Université Laval, Québec, QC G1V 0A6, Canada; 4 Centre Nutrition, Santé et Société (NUTRISS), Institute of Nutrition and Functional Foods (INAF), Université Laval, Québec, QC G1V 0A6, Canada; marie-claude.vohl@fsaa.ulaval.ca

**Keywords:** cholestatic autoimmune liver diseases, primary biliary cholangitis, primary sclerosing cholangitis, ursodeoxycholic acid, omega-3 polyunsaturated fatty acids, combination therapy, bile acid metabolism and toxicity, ER stress, inflammation

## Abstract

Ursodeoxycholic acid (UDCA) is the first line therapy for the treatment of cholestatic and autoimmune liver diseases. Its clinical use is currently limited by a significant proportion of non-responder patients. Polyunsaturated fatty acids (n-3 PUFAs) possess important anti-inflammatory properties and protect liver cells against bile acid (BA)-induced toxicity. The present study was designed to rapidly evaluate whether combining n-3 PUFAs (i.e., eicosapentaenoic [EPA] and docosahexaenoic [DHA] acids) to UDCA would provide additional benefits when compared to the drug alone. The parameters evaluated were (i) the expression of genes governing BA synthesis, transport, and metabolism; (ii) the prevention of BA-induced apoptosis and endoplasmic reticulum (ER)-stress; and (iii) the control of BA- and LPS-dependent inflammation. In the absence of n-3 PUFAs, most of the parameters investigated were unaffected by UDCA or were only altered by the higher dose (500 µM) of the drug. By contrast, in the presence of EPA/DHA (50/50 µM), all parameters showed a strongly improved response and the lowest UDCA dosage (50 µM) provided equal or better benefits than the highest dose used alone. For example, the combination EPA/DHA + UDCA 50 µM caused comparable down-regulation of the *CYP7A1* gene expression and of the BA-induced caspase 3 activity as observed with UDCA 500 µM. In conclusion, these results suggest that the addition of n-3 PUFAs to UDCA may improve the response to the drug, and that such a pharmaco-nutraceutical approach could be used in clinic to open the narrow therapeutic dose of UDCA in cholestatic liver diseases.

## 1. Introduction

Ursodeoxycholic acid (UDCA) is the most widely used therapy for several liver disorders, including chronic cholestatic and autoimmune liver diseases such as primary biliary cholangitis (PBC) and primary sclerosing cholangitis (PSC) [1,2,3,4]. Initially, UDCA was thought to reduce the toxicity of the bile acid (BA) pool by replacing the hydrophobic acids by more hydrophilic and less toxic derivatives [5]. However, experimental evidences now suggest that the benefits of UDCA are obtained through a range of more complex actions such as choleretic and anti-apoptotic effects, as well as immunoregulatory properties [2,6,7]. Accordingly, UDCA increases biliary secretion of bile acids and HCO_3_^-^ and also modulates the expression of BA-detoxification related genes [8,9,10,11]. Furthermore, UDCA therapy reduces levels of apoptotic cells, inflammatory and fibrotic markers (TNF-α and TGF-β), while inhibiting the overexpression of hepatic MHC-I and adhesion molecules in immune and biliary cells in PBC patients [12,13,14,15,16,17,18]. Despite these multiple mechanisms of action, the therapeutic benefits of UDCA remain to be improved. Indeed, approximately 30 to 40% of PBC patients do not respond or are intolerant to the drug [3,19]. In PSC, UDCA might improve liver biochemistry but remains ineffective in influencing the frequency of death or liver transplantation outcomes [20,21]. At high dose, UDCA was associated with an increased rate of serious adverse events [20,21]. The unique FDA-approved alternative to UDCA, namely obeticholic acid (OCA), exerts beneficial effects in PBC and PSC treatment [22], but the occurrence of important side effects (such as pruritus), also limits its clinical use. Thus, important pharmacological needs remain unmet in the treatments of these chronic cholestatic conditions [3,4,19,22]. However, given the benefits of UDCA therapy in responsive PBC patients and its accessibility, pharmacological strategies aimed at increasing the effectiveness of the drug (such as combination therapies) could rapidly lead to the implementation of better treatments for PBC and PSC.

Omega-3 polyunsaturated fatty acids (n-3 PUFAs), namely eicosapentaenoic acid (EPA) and docosahexaenoic acid (DHA), exert multiple beneficial effects in various chronic diseases such as cardiovascular and neurodegenerative conditions, rheumatoid arthritis, hypertriglyceridemia, and cancer [23]. N-3 PUFAs are well-known for their diverse anti-inflammatory properties: namely, increased production of anti-inflammatory molecules (eicosanoids, endocannabinoids, resolvins, and protectins), inhibition of cytokine production, reduction of NF-κB activation, down-regulation of surface adhesion molecules, reduction of leukocyte chemotaxis, and a decrease in T-cell reactivity (reviewed in [24]). Several evidences support the therapeutic potential of n-3 PUFAs (mainly DHA and EPA) on metabolic liver diseases such as non-alcoholic fatty liver disease (NAFLD) and/or non-alcoholic steatohepatitis (NASH) (reviewed in [25]). EPA and DHA also exert protective effects against BA-induced cell death in human hepatocytes [26,27,28]. This effect could be attributed, at least in part, to their capacity to stimulate BA detoxification, by inhibiting their synthesis and stimulating their metabolism [26]. Finally, the idea that n-3 PUFA may have a place in the pharmacological armamentarium of chronic cholestatic liver diseases has emerged after the observation that DHA significantly reduced alkaline phosphatase (ALP) levels in PSC patients [29]. In this study—as the reduction of ALP levels remained relatively moderate [29]—instead of using n-3 PUFAs as the main therapeutics for cholestatic liver diseases, we sought to examine whether EPA and DHA could be used to improve the effectiveness of other anticholestatic therapies, such as UDCA. Such an hypothesis is supported by various investigations previously revealing that n-3 PUFAs can be efficiently used to improve the response to other drugs, such as 17β-estradiol [30] or retinoic acids [31].

The present study therefore investigates whether n-3 PUFAs and UDCA combinations could provide superior responses compared to UDCA alone with regards to (1) BA detoxification, (2) BA-induced toxicity, and (3) inflammation in human HepG2 cells and THP-1 macrophages.

## 2. Materials and Methods

### 2.1. Cell Culture

HepG2 and THP-1 cell lines were obtained from the American Type Culture Collection (Manassas, VA, USA). Cell culture media, FBS, penicillin/streptomycin, nonessential amino-acids, and other cell culture reagents were purchased from Wisent (Québec, QC, CA). Cells were cultured in Dulbecco’s modified Eagle’s medium (DMEM) supplemented with 10% FBS, 1% L-glutamine, penicillin/streptomycin, and nonessential amino-acids. All experiments were performed in serum-free medium.

For RNA analyses, HepG2 cells were plated in 12-well plates (200,000 cells per well) and cultured in the presence of DMSO (vehicle, 0.1% *v/v*), EPA/DHA (50 µM), UDCA (50–500 µM), or a combination of EPA/DHA/UDCA at indicated concentrations for 24 h.

For apoptosis studies, HepG2 cells (175,000 cells per well, 24-well plates) were pretreated for 24 h with vehicle (DMSO/ethanol) or EPA/DHA (50 µM each) and UDCA (50–500 µM) individually or in combination at the indicated concentrations. A BA mixture composed of 100 µM of CA, CDCA, LCA, and DCA, was added for 2 h prior to caspase-3 activity measurement.

For ER stress experiments, HepG2 cells were seeded in 12-well plates and treated for 24 h with the same BA mixture as above, in the absence or presence of EPA/DHA (50/50 µM), UDCA (50–500 µM) or the EPA/DHA + UDCA combinations.

For inflammation studies, THP-1 cells (1,000,000 cells per well, 6-well plates) were first differentiated into macrophages in the presence of 100 nM PMA for 72 h. Following differentiation, TPH-1 differentiated macrophages were stimulated with 100 ng/mL LPS (Sigma, St-Louis, MO) for 24 h in the presence or absence of either EPA/DHA (50 µM), UDCA (50–500 µM) or a combination.

### 2.2. RNA Isolation, Reverse Transcription and Quantitative Real-Time Polymerase Chain Reaction (qRT-PCR)

Total RNA was isolated using the Tri-Reagent^®^ protocol as recommended by the manufacturer (Molecular Research Center Inc., Cincinnati, OH, USA). cDNA was obtained by reverse transcription (RT) reactions with 1µL of SuperScript™ IV Vilo™ master mix (Thermo Scientific, Life Technologies Division, Carlsbad, CA, USA) and 500 ng of isolated RNA in a final reaction volume of 5 µL. Gene mRNA expression was detected using Fast SYBR^®^ Green real-time polymerase chain reaction master mix (Thermo Scientific, Life Technologies Division, Carlsbad, CA, USA) in an ABI ViiA7 system (Applied Biosystems, Foster City, CA, USA). Each reaction was performed in a final volume of 10 µL containing 5 µL of SybrFast^®^ PCR mix, 1 µL of forward and reverse primers [26], and 3 µL of diluted RT product. qRT-PCR reactions were carried out at 95 °C for 20 s, 95 °C for 30 s, and annealing temperature for 20 s for 40 cycles. Threshold cycle (Ct) values were analyzed using the comparative Ct (ΔΔCt) method as recommended by the manufacturer (Thermo Scientific, Waltham, MA, USA). Target gene mRNA levels were obtained by normalizing to the endogenous reference Pumilio RNA-Binding Family Member 1 (*PUM1*) and expressed relatively to vehicle-treated cells set at 1.

### 2.3. Caspase-3 Assay

Caspase-3 activity was determined using the Enzcheck^®^ caspase-3 assay kit (Thermo Scientific, Life Technologies Division, Carlsbad, CA, USA). Assays were performed according to the manufacturer’s instructions. Mean fluorescence was measured with an Infinite M1000 instrument (Tecan, Austria). Results are expressed as mean fluorescence normalized by sample protein concentration as determined by BCA assay (Bio-rad Laboratories, Hercules, CA, USA). Caspase-3 activity levels are expressed relative to vehicle-treated cells set at 1.

### 2.4. ELISA

IL-6 and TNF-α secretion from THP-1 cells were determined using Human IL-6 DuoSet^®^ (R&D Systems, Minneapolis, MN, USA) and Human TNF-α (Invitrogen, Carlsbad, CA, USA) ELISA kits following the manufacturers’ instructions. Cytokine production was expressed relatively to vehicle treated cells.

### 2.5. Statistics

Differences in cell responses between treatments were determined by one-way ANOVA followed by Tukey’s multiple comparison test post-hoc. *p* values < 0.05 were considered statistically significant. Statistical analyses were performed using Graph-Pad Prism version 7.0 (GraphPad Software, La Jolla, CA, USA, http://www.graphpad.com, accessed on 15 May 2020). Reported *p*-values for treatment groups comparisons are multiplicity adjusted *p* values.

## 3. Results

### 3.1. N-3 Polyunsaturated Fatty Acids Improve the Transcriptional Signature of UDCA in HepG2 Cells

**Figure 1:**
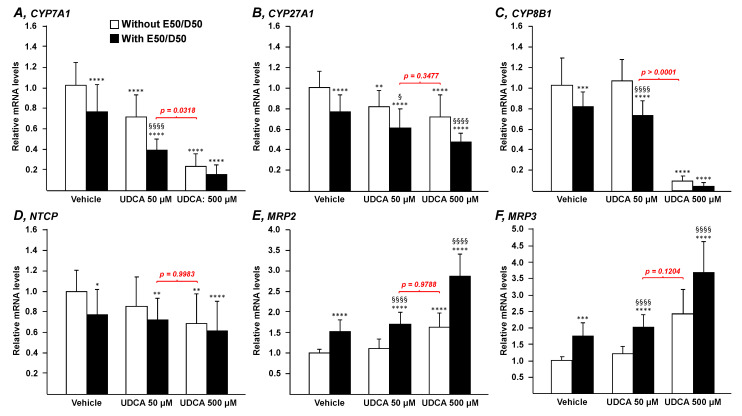
N-3 PUFAs potentiate the effects of UDCA on genes governing bile acid homeostasis. Human hepatoma HepG2 cells were treated with vehicle (DMSO), EPA/DHA (50/50 µM), UDCA (50 or 500 µM) or a combination of EPA/DHA (50/50 µM) and UDCA (50 or 500 µM) for 24 h. Total RNA was extracted. *CYP7A1* (**A**), *CYP27A1* (**B**), *CYP8B1* (**C**), *NTCP* (**D**), *MRP2* (**E**), and *MRP3* (**F**) transcript levels were quantified by qRT-PCR as detailed in the materials and methods section, and mRNA levels were expressed relatively to control cells set at 1. Data represent the mean of 3 independent experiments in which each treatment was performed in quadruplicate. Each data point therefore corresponds to the mean of 12 replicates ± SD. Statistical significances as determined by a one-way ANOVA followed by Tukey’s multiple comparison post-hoc were as follows: Vehicle vs. UDCA treated cells: * *p* < 0.05; ** *p* < 0.01, *** *p* < 0.001, **** *p* < 0.0001; UDCA vs. UDCA + EPA/DHA: § *p* < 0.05, §§§§ *p* < 0.0001 E50:D50: EPA 50 µM and DHA 50 µM.

To determine whether n-3 PUFAs impact the ability of UDCA to modulate the expression of essential genes for the control of bile acid synthesis (*CYP7A1, CYP27A1,* and *CYP8B1*), uptake (*NTCP*), or export (*MRP2* and *MRP3*), HepG2 cells were exposed to UDCA (50 or 500 µM) in the presence or absence of EPA/DHA (50/50 µM) for 24 h (Figure 1).

In the absence of the n-3 PUFAs fatty acids (Figure 1; white bars), UDCA dose-dependently decreased *CYP7A1* and *CYP27A1* mRNA levels (Figure 1A,B). *CYP8B1*, *NTCP*, and *MRP2* transcripts were significantly affected only in the presence of the highest UDCA dose (Figure 1C–E), while *MRP3* mRNA levels remained unchanged (Figure 1F).

In the presence of 50 µM EPA/DHA (Figure 1; black bars), most of the responses to UDCA were improved. When added to UDCA 50 µM, EPA and DHA significantly (*p* < 0.0001) increased the reduction rate of *CYP7A1* mRNA levels from 29% (w/o EPA/DHA) to 60% (with EPA/DHA) (Figure 1A). A similar—but less spectacular—improvement was also observed with the expression of the *CYP27A1* gene (*p* < 0.05) (Figure 1B). In the presence of 500 µM UDCA, similar improvements were also observed but the statistical significance was reached only for *CYP27A1* (*p* < 0.001). EPA/DHA addition to 50 µM UDCA also resulted in significant changes in the expression of *CYP8B1, NTCP, MRP2,* and *MRP3*, a response that was not achieved with 50 µM UDCA alone (Figure 1C–F). The additive effect of EPA/DHA was maintained in the presence of 500 µM UDCA, but only for *MRP2* and *MRP3* (*p* < 0.0001) (Figure 1E,F). Overall, these data indicate that, when combined, UDCA and n-3 PUFAs exert additive effects on the expression of the genes.

The most interesting observation issuing from these experiments concerns the lack of statistical significance observed when comparing mRNA levels in cells cultured in the presence of the combination 50 µM UDCA plus EPA/DHA *versus* those from cells exposed to 500 µM UDCA alone. Indeed, with the exception of *CYP8B1* (Figure 1C), all genes responded to the combination UDCA 50 µM and EPA/DHA in a very similar manner as to UDCA 500 µM (Figure 1; *p* value in red), even if in the case of *CYP7A1*, the difference remained statistically significant (*p* = 0.0318; Figure 1A). This last observation reveals that not only does the addition of EPA/DHA 50 µM improve the response to a low dose of UDCA in terms of BA related transcriptomic signature, but that such improvement also leads to the same response as that obtained in the presence of a 10-times-higher Urso dose.

### 3.2. N-3 Polyunsaturated Fatty Acids Improve the Hepatoprotective Effects of Ursodeoxycholic Acid against BA-Induced Apoptosis in HepG2 Cells

Bile acid accumulation in the liver activate cell death pathways, thus contributing to the development of liver damage under cholestatic conditions [32]. Because both UDCA and n-3 PUFAs were reported as hepatoprotective molecules against BA-induced toxicity [26,32], we sought to determine whether their combination could provide superior protection against BAs compared to UDCA alone. For this purpose, HepG2 cells were treated with UDCA (50 or 500 µM) alone or in the presence of EPA/DHA (50/50 µM) for 24 h, then exposed to a toxic BA mixture (CA, CDCA, LCA, and DCA, 100 µM each) for an additional 2 h prior to being subjected to a measurement of caspase-3 activity as a means to evaluate the extent of cell death.

**Figure 2:**
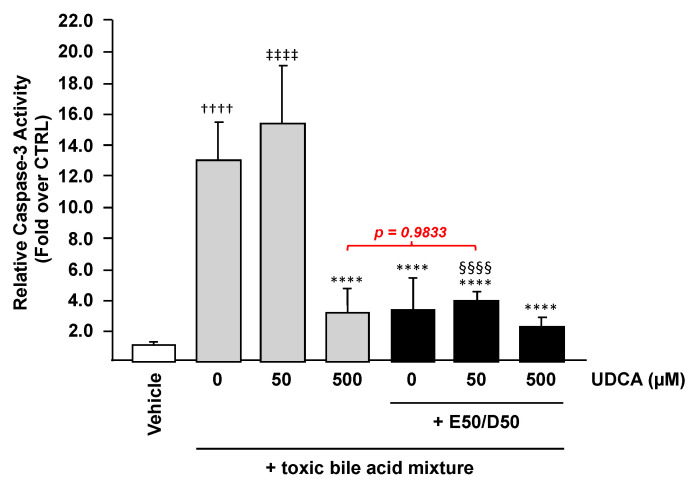
EPA and DHA protect liver cells against bile acid-induced apoptosis in the presence of UDCA. HepG2 cells were treated with vehicle (DMSO), EPA/DHA, UDCA, or a combination of EPA/DHA + UDCA at the indicated doses for 24 h, and subsequently exposed for 2 h treatment to the vehicle or a toxic BA mixture (CA, CDCA, LCA, and DCA, 100 µM each), caspase 3 activity was then quantified as detailed in the materials and methods section and expressed relatively to control cells set at 1. Data represent the mean of two independent experiments in which each treatment was performed in quadruplicate. Each data point therefore corresponds to the mean of 8 replicates ± SD. Statistical significances as determined by a one-way ANOVA followed by Tukey’s multiple comparison post-hoc were as follows: Untreated cells exposed to vehicle vs. untreated cells exposed to bile acids: †††† *p* < 0.0001; Vehicle cells vs. UDCA treated cells: ‡‡‡‡ *p* < 0.0001; BA exposed cells vs. BA + UDCA ± EPA/DHA exposed cells: **** *p* < 0.0001; UDCA treated cells vs. UDCA + EPA/DHA treated cells: §§§§ *p* < 0.0001; E50:D50: EPA 50 µM and DHA 50 µM.

As expected [26], cells exposed to the BA mixture exhibited a 12-fold higher caspase-3 activity (*p* < 0.0001) when compared to vehicle-exposed cells (Figure 2). In the absence of EPA/DHA, 50 µM UDCA failed to inhibit the BA-induced caspase-3 activation, while at high concentration (500 µM) the drug was efficient in reducing the elevated caspase activity caused by exposure to the BA mixture (Figure 2). A similar attenuation of the BA-induced caspase-3 activity was observed as soon as cells were pre-treated with n-3 PUFAs with or without UDCA (Figure 2). Consequently, and as observed with mRNA levels, these data demonstrate that the addition of EPA/DHA to a low UDCA dose improves the drug response and allows a low dose of the anti-cholestatic drug to prevent BA-induced cell death.

### 3.3. N-3 Polyunsaturated Fatty Acids Suppress the BA-Induced Endoplasmic Reticulum Stress and Inflammation in HepG2 Cells

Considering that ER stress-induced apoptosis and secretion of inflammatory cytokines such as IL-8 are also involved in the progression of cholestatic liver injury [33,34,35,36], we next investigated whether n-3 PUFAs and UDCA can exert additive effects on mRNA expression levels of BA-induced ER-stress marker BiP, ER-stress mediated apoptosis marker CHOP and the inflammatory marker IL-8 in HepG2 cells.

**Figure 3:**
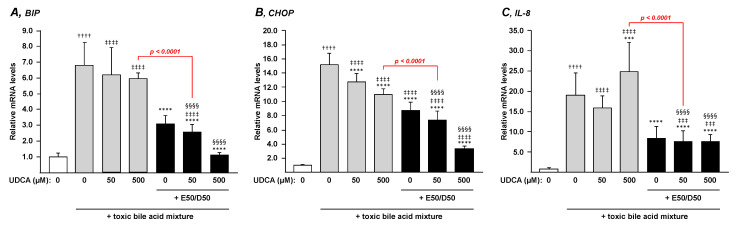
N-3 PUFAs strongly improve the ability of low and high UDCA doses to prevent ER stress and inflammation. HepG2 cells were exposed to vehicle (DMSO) or a toxic BA mixture (i.e., CA, CDCA, LCA and DCA, 100 µM each) for 24 h in the absence or presence, of EPA/DHA (50/50 µM), UDCA (50–500 µM), or the EPA/DHA + UDCA combinations. Total RNA was extracted and analyzed for transcript levels of the ER-stress BIP (**A**) and CHOP (**B**) and inflammatory IL-8 (**C**) markers by qRT-PCR as detailed in the materials and methods section, and mRNA levels were expressed relatively to control cells set at 1. Data represent the mean of two independent experiments in which each treatment was performed in quadruplicate. Each data point therefore corresponds to the mean of 8 replicates ± SD. Statistical significances as determined by a one-way ANOVA followed by Tukey’s multiple comparison post-hoc were as follows: Untreated cells exposed to vehicle vs. untreated cells exposed to BA: †††† *p* < 0.0001; Vehicle cells vs. UDCA treated cells: ‡‡‡ *p* < 0.001; ‡‡‡‡ *p* < 0.0001; BA exposed cells vs. BA + UDCA ± EPA/DHA exposed cells: *** *p* < 0.001; **** *p* < 0.0001; UDCA treated cells vs. UDCA + EPA/DHA treated cells: §§§§ *p* < 0.0001.

The same toxic BA mixture as above caused a marked increase in mRNA expression of *BiP*, *CHOP,* and *IL-8* in HepG2 cells (Figure 3A–C). While neither low nor high UDCA doses caused any changes in *BiP* or *IL-8* mRNA levels (Figure 3A,C), *CHOP* transcripts were dose-dependently reduced (*p* < 0.0001) by UDCA (Figure 3B). EPA/DHA alone caused significant reductions of the BA-dependent induction of *BIP*, *CHOP,* and *IL-8* mRNA levels (Figure 3). The addition of low or high UDCA doses failed to provide any additional benefits to the response of EPA/DHA on *BiP* and *IL-8* mRNA levels (Figure 3A,C). By contrast, UDCA dose-dependently improved (*p* < 0.0001) the ability of EPA/DHA to limit the BA-dependent activation of *CHOP* mRNA induction (Figure 3B).

The benefits of n-3 PUFAs are such that for the 3 genes, when combined to EPA/DHA, the lower UDCA dose provided better prevention against BA-induced ER stress and inflammation than the highest UDCA dose used alone (Figure 3; *p* value in red). This last observation indicates that the combination of EPA/DHA + low UDCA dose is more effective than UDCA alone in preventing ER stress and inflammation.

### 3.4. The Combination n-3 PUFAs Plus UDCA Suppresses the LPS-Dependent Induction of Pro-inflammatory Mediators in THP-1 Macrophages

Both UDCA and n-3 PUFAs exhibit anti-inflammatory properties [6,24], thus we next evaluated whether, when combined, UDCA and EPA/DHA conserve their anti-inflammatory effects in LPS-stimulated THP-1 macrophages.

**Figure 4:**
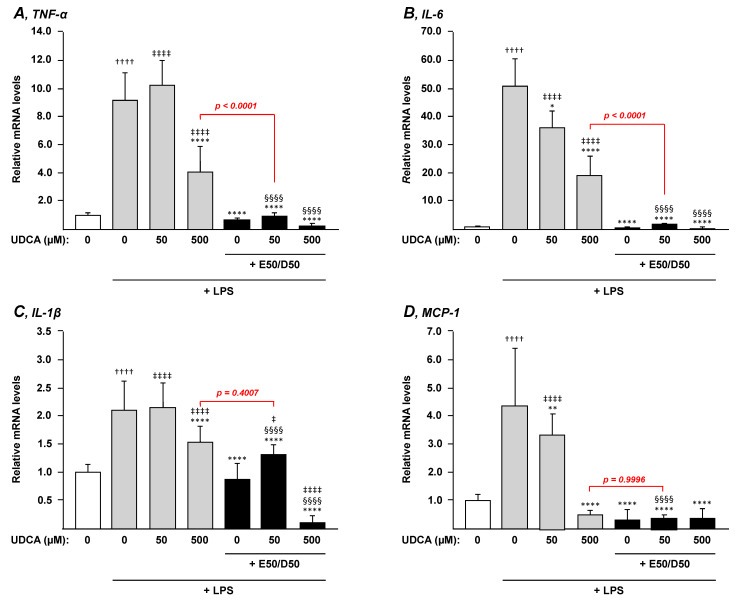
Addition of n-3 PUFAs to UDCA increases treatment anti-inflammatory potential in macrophages. THP-1 monocytes were differentiated into macrophages with PMA for 72 h and then stimulated with 100 ng/µL LPS for 24 h in the presence or absence of EPA/DHA, UDCA, or their combination as described in the materials and methods section. Total RNA was extracted. TNF-α (**A**), IL-6 (**B**), IL-1β (**C**), and MCP-1 (**D**) transcript levels were quantified by qRT-PCR as detailed in the materials and methods section, and mRNA levels were expressed relatively to control cells (i.e., without LPS challenge) set at 1. Data represent the mean of 3 independent experiments in which each treatment was performed in triplicate. Each data point therefore corresponds to the mean of 9 replicates ± SD. Statistical significances as determined by a one-way ANOVA followed by Tukey’s multiple comparison post-hoc were as follows: Untreated differentiated THP-1 exposed to vehicle vs. untreated differentiated THP-1 exposed to LPS: †††† *p* < 0.0001; Vehicle differentiated THP-1 vs. UDCA treated differentiated THP-1: ‡‡‡‡ *p* < 0.0001; LPS exposed differentiated THP-1 vs. LPS + UDCA ± EPA/DHA exposed differentiated THP-1: * *p* < 0.05; ** *p* < 0.01; **** *p* < 0.0001; UDCA treated differentiated THP-1 vs. UDCA + EPA/DHA treated differentiated THP-1: §§§§ *p* < 0.0001.

As illustrated in Figure 4, mRNA levels of the inflammation markers *TNF-α*, *IL-6*, *IL-1β* and *MCP-1* were markedly increased in PMA-differentiated THP-1 macrophages exposed to LPS. While 500 µM UDCA significantly reduced mRNA levels of all markers in these cells (*p* < 0.0001) (Figure 4A–D), only *IL-6* and *MCP-1* mRNA levels exhibited a dose-dependent pattern with significant-but less spectacular-reductions in the presence of the lower UDCA dose (Figure 4B,D). Interestingly, the EPA/DHA treatment totally (*p* < 0.0001) abolished the LPS-dependent activation of *TNF-α, IL-6, IL-1β* and *MCP-1* mRNA expression (Figure 4). These inhibitory effects were preserved in the presence of UDCA (low and high doses) (Figure 4). Consequently, the combination EPA/DHA and UDCA (50 µM) caused a stronger reduction in all markers when compared to the high dose of UDCA (500 µM), thus suggesting that the addition of EPA/DHA to low UDCA dosage provides a better advantage than high UDCA. Accordingly, and as highlighted with red *p values*, the combination of 50 µM UDCA and EPA/DHA caused either a similar (IL-1β and MCP-1, Figure 4C,D) or a stronger (*p* < 0.0001; TNF-α and IL-6, Figure 4A,B) reduction in transcript levels than with 500 µM UDCA.

**Figure 5:**
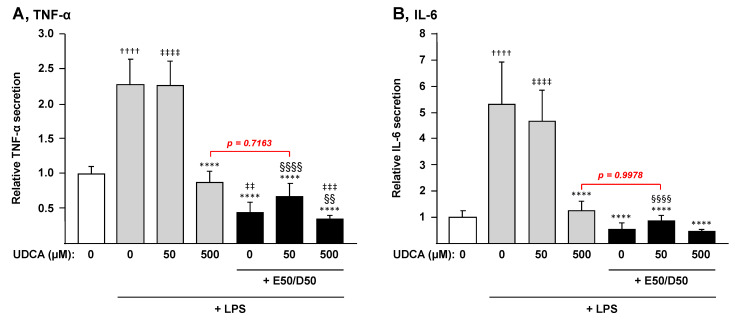
N-3 PUFAs block LPS-induced secretion of cytokines by macrophages. THP-1 monocytes were differentiated into macrophages with PMA for 72 h and then stimulated with 100 ng/µL LPS for 24 h in the presence or absence of EPA/DHA, UDCA or their combination. The level of IL-6 and TNF-α cytokines in culture media was measured by ELISA as described in the materials and methods section. Cytokine levels are expressed relatively to control cells set at 1. Data represent the mean of two independent experiments in which each treatment was performed in triplicate. Each data point therefore corresponds to the mean of 6 replicates ± SD. Statistical significances as determined by a one-way ANOVA followed by Tukey’s multiple comparison post-hoc were as follows: Untreated differentiated THP-1 exposed to vehicle vs. untreated differentiated THP-1 exposed to LPS: †††† *p* < 0.0001; Vehicle differentiated THP-1 vs. UDCA treated differentiated THP-1: ‡‡ *p* < 0.01; ‡‡‡ *p* < 0.001; ‡‡‡‡ *p* < 0.0001; LPS exposed differentiated THP-1 vs. LPS + UDCA ± EPA/DHA exposed differentiated THP-1: **** *p* < 0.0001; UDCA treated differentiated THP-1 vs. UDCA + EPA/DHA treated differentiated THP-1: §§ *p* < 0.01; §§§§ *p* < 0.0001.

We next sought to determine whether these effects on mRNA levels also resulted in changes in levels of cytokines secreted by macrophages (Figure 5). For this purpose, the secretion of TNF-α and IL-6 in the cell media was quantified by ELISA. These analyses mainly confirmed the mRNA observations revealing that combining EPA + DHA to UDCA 50 µM drastically improves the UDCA-dependent prevention of cytokine secretion and leads to a similar reduction as that in the presence of a 10-fold higher concentration of the drug (Figure 5).

Altogether, addition of EPA/DHA to low UDCA treatments confers a very efficient protection against LPS-induced expression of pro-inflammatory mediators.

## 4. Discussion

**Figure 6:**
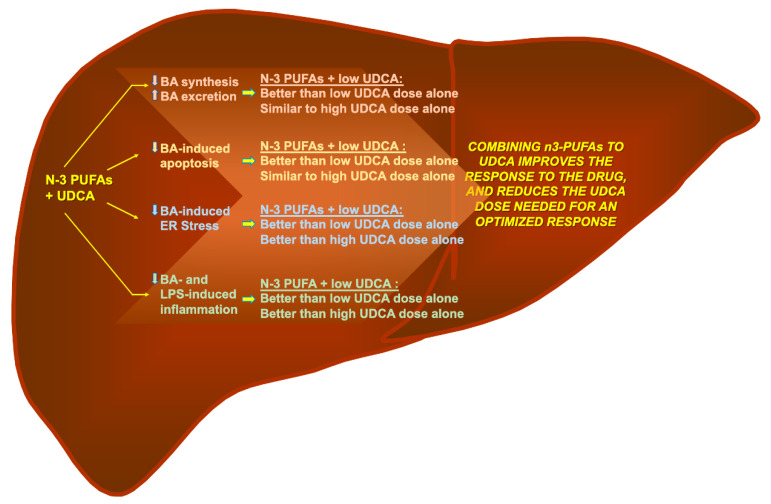
Omega-3 polyunsaturated fatty acid: a pharmaco-nutraceutical approach to improve the responsiveness to ursodeoxycholic acid. Results from the present study indicate that n-3 polyunsaturated fatty acids (n-3 PUFAs), such as EPA and DHA, improve the response of low UDCA dosage with regards to the reduction of BA toxicity, the prevention of BA-induced apoptosis and ER-stress, as well as the reduction of BA- or LPS-induced inflammation. These data support the idea that combining n-3 PUFAs with UDCA improves the response to the drug and reduces the UDCA dose required for an optimized response.

The present study reveals that the addition of EPA and DHA ameliorates the response to both low and high UDCA doses in human cell models. These improvements were observed in terms of gene expression related to BA-detoxification, inhibition of BA-induced apoptosis and suppression of ER stress, as well as inflammatory markers. Interestingly, for most if not all parameters analyzed, the combination of EPA/DHA and a low (50 µM) UDCA dose led to similar or improved responses to those observed with the elevated (500 µM) dosage. Actually, for most parameters used to evaluate apoptosis and inflammation the lowest UDCA doses remained ineffective, while n-3 PUFAs alone caused solid improvements. Thus, it can be envisioned that most of the benefits of combining n-3 PUFAs and UDCA on these parameters are due to the fatty acids. Nevertheless, these observations support the idea that the addition of n-3 PUFAs, EPA and DHA, to UDCA may provide clinical benefits in terms of improved response to and optimized dosage of UDCA (Figure 6).

To the best of our knowledge, we present here the first evidence that a combination EPA/DHA + UDCA could be beneficial for the treatment of cholestatic liver diseases. UDCA is the first line therapy for numerous cholestatic liver diseases, but its clinical usage still suffers some limitations, at least for the treatment of the cholestatic and autoimmune liver diseases PBC and PSC. Indeed, up to 40% of PBC patients do not respond adequately to UDCA, with 5–10% who are intolerant to the drug [3,19], while at the recommended therapeutic dosages its effectiveness to prevent liver failure in PSC patients is questionable and the use of high doses of UDCA is associated with serious adverse events [20,37]. Among the strategies that were evaluated to improve the responsiveness to UDCA, several drug combinations were assayed. Because the first mechanism of liver injury in PBC is immune-mediated inflammation of hepatobiliary cells, immunosuppressive treatments were the first to be assessed, and shown to be ineffective alone and in adjunctive therapies (Reviewed in [38]). A 3-year randomized, but open label, clinical study revealed that only 25% of patients receiving the combination of budesonide with UDCA showed regression in liver fibrosis [39]. As fully discussed in Chascsa and Lindor 2020 [38], UDCA combination with other immunosuppressive agents, such as mycophenolate, methotrexate, or glucocorticoids (i.e., budesonide or prednisone), also showed conflicting results, with low or no significant clinical or liver biochemical benefits [38]. Similar observations were also reported in the limited number of trials investigating the combination of UDCA and immunosuppressive agents in PSC patients (reviewed in [40]). The absence of clear benefits are thought to reflect the fact that immunosuppressive treatments often lack specific targets [38]. Beyond immunosuppressive drugs, a series of other treatments were also tested for combination with UDCA (reviewed in [38]); the most promising UDCA combination therapies involve fibrates, such as fenofibrate and bezafibrate (reviewed in [38]). Fibrates (i.e., fibric acid derivatives) are triglycerides lowering and anti-inflammatory drugs acting through activation of nuclear receptors belonging to the peroxisome proliferator-activated receptor (PPAR) subfamily [41]. Very recently, Ghonem and colleagues [42] reported that, when added to UDCA in PBC and PSC patients with incomplete responses, fenofibrate caused a significant reduction, and in some cases normalization, of liver enzymes as well as pro-inflammatory cytokines and reduced the toxicity of bile acids [38,42]. Similarly, a clinical trial showed that the UDCA and bezafibrate combination was, by far, more efficient than UDCA alone in achieving a complete biochemical response as defined by surrogate markers including bilirubin, ALP, albumin, and prothrombin index time (PT) in PBC patients [43]. Together, these observations suggested that combining a PPAR-alpha agonist to UDCA would be helpful in treating PBC and/or PSC. However, the increased occurrence of creatinine elevation and myalgia in the bezafibrate group prompted the authors to conclude that advanced cirrhosis and severe cholestasis could limit therapy with bezafibrate [38,43]. Similarly, another study with fenofibrate confirmed the clear benefit of the drug with improved transplant-free and overall survival, but also evidenced that cirrhotic patients had a more rapid increase in serum bilirubin, suggesting a propensity of fenofibrate to precipitate hepatic decompensation in patients with advanced fibrosis [38,44]. Together these observations suggest that even if PPAR-alpha activation is considered as an interesting therapeutic avenue to combine with UDCA, these side effects may limit the use of fibrates.

N-3 PUFAs, such as EPA and DHA, are PPAR activators, and they exert similar effects to fibrates on triglycerides reduction [41,45], BA detoxification [26,46], and the control of pro-inflammatory cytokines production [41,47]. Thus, while future functional investigations are needed to fully decipher the molecular mechanisms of UDCA and PUFAs on gene expression levels, one can speculate that the PPARα receptor may play a role, at least in mediating the PUFAs’ effects. The present study therefore suggests that N-3 PUFAs such as EPA and DHA may present a strong alternative to fibrates in combination therapies involving a PPAR activator and UDCA. As indicated earlier, DHA was recently tested in PSC patients, and was found effective in reducing serum ALP levels, without any significant side effects reported [29]. It is therefore tempting to speculate that the combination of EPA/DHA and UDCA may provide therapeutic benefits in PBC and PSC treatment. While additional in vivo or clinical studies are required to validate this hypothesis, recent investigations revealed that UDCA and n-3 PUFAs synergize to provide superior outcomes than individual treatments in gallstone dissolution and in NASH resolution in mice [48,49]. These last observations support the idea that these in vitro improvements of UDCA-induced responses by n-3 PUFAs reported here will remain in future in vivo studies with PBC and PSC animal models. Mice strains such as the NOD.c3c4 (PBC) or Mdr2^−/−^ (PSC) are classical models used for studies aimed at identifying novel treatments for cholestatic and autoimmune liver. Such experimental models would not only be useful to validate the full transcriptomic signature of n-3 PUFAs + UDCA combination, but they are also needed to validate the therapeutic potential of such treatment by demonstrating their antifibrotic effects, for example.

As illustrated in Figure 6, not only does the addition of EPA and DHA improve the impact of UDCA on several parameters targeted by the drug (i.e., reduction of *CYP7A1* and *CYP27A1* gene expression levels and LPS-induced cytokines production), but also provided additive benefits that were absent with the drug alone; namely increased *MRP2/3* expression and reduced expression of BA-induced ER-stress markers. Previous investigations from our lab revealed the dose-dependent manner in which EPA and/or DHA regulates the mRNA expression of bile acid-related genes such as *CYP7A1*, *CYP27A1*, and *MRP2/3* [26]. For all genes, the optimal response was obtained in the presence of 50 μM EPA plus 50 μM DHA, thus it can be anticipated that lower n-3 PUFAs doses would be less efficient to modulate UDCA efficacy. The fact that UDCA alone was inefficient in lowering ER-stress markers was intriguing since previous studies reported that it and its taurine conjugates, TUDCA, were able to reduce ER-stress markers *BIP* and *CHOP* in hepatocytes and cholangiocytes (biliary epithelial cells) activated by the prototypical ER-stress inducers [35,50,51]. These differences may relate to different experimental settings. Nonetheless, the fact that the EPA/DHA treatment efficiently reduces *BiP* and *CHOP* expression in the presence, and even in the absence, of UDCA is of particular clinical interest since ER-stress and ER-stress mediated apoptosis is a common feature of cholestatic liver diseases [33,34,35,36].

Beyond the improvement of the response to UDCA, one of the most interesting observations revealed from the present study relates to the fact that adding n-3 PUFAs allows a significant reduction of the UDCA dose required to reach an optimal response in terms of BA-synthesizing and metabolizing gene expression. Indeed, and as extensively discussed in [38], the therapeutic dose of UDCA is very narrow. For PBC patients, lower doses of UDCA (5–7 mg/kg/day) are inferior to the recommended 13–15 mg/kg/day dosing, while 23–25 mg/kg/day dosages do not offer any additional clinical benefit [38]. Higher doses, namely 28–30 mg/kg/day have been shown to be inadequate for PSC patients with an increase in hepatic decompensation [37,38]. It has therefore been envisioned that when combined to EPA and DHA, a low to normal dose of UDCA may offer the same benefits as the high dosage, but without the deleterious side effects.

In conclusion, while suffering some limitations due to its descriptive nature and the use of immortalized human cell models, the present study provides strong evidence of the benefits that a combination of EPA/DHA and UDCA may exert in controlling factors such as BA metabolism, BA-induced apoptosis, ER-stress, and inflammation. These data provide a solid basis for the design of additional in vivo and clinical investigations which, hopefully, will demonstrate that the addition of EPA and DHA as a pharmaco-nutraceutical approach may improve the responsiveness to UDCA in cholestatic liver diseases, and potentially any liver diseases that may have increased hepatocellular damage and ER stress. These future investigations are particularly needed to validate the idea that n-3 PUFAs potentiate the effects of UDCA on bile acid metabolism.

## Data Availability

Data could be provided on demand to the corresponding author.
